# The genomic and immune landscapes of gastric cancer and their correlations with HER2 amplification and PD‐L1 expression

**DOI:** 10.1002/cam4.6765

**Published:** 2023-12-05

**Authors:** Xiaoqian Jing, Zhiping Luo, Jiayan Wu, Feng Ye, Jianfang Li, Zijia Song, Yaqi Zhang, Minmin Shi, Huaibo Sun, Yi Fang, Yimei Jiang, Xiaopin Ji

**Affiliations:** ^1^ Department of General Surgery Ruijin Hospital affiliated to Shanghai Jiao Tong University School of Medicine Shanghai China; ^2^ Genecast Biotechnology Co., Ltd Wuxi Jiangsu China; ^3^ Department of Surgery, Shanghai Key Laboratory of Gastric Neoplasms Shanghai Institute of Digestive Surgery, Ruijin Hospital affiliated to Shanghai Jiao Tong University School of Medicine Shanghai China; ^4^ Research Institute of Pancreatic Diseases affiliated to Shanghai Jiao Tong University School of Medicine Shanghai China; ^5^ Department of Emergency Shanghai Tenth People's Hospital Shanghai China

**Keywords:** gastric cancer, genomic landscape, HER2 amplification, immune landscape, PD‐L1

## Abstract

**Background:**

Anti‐PD1/PD‐L1 antibody plus human epidermal growth factor receptor 2 (HER2) antibody and chemotherapy have become the new first‐line therapy for HER2 overexpression‐positive advanced gastric cancers (GC), suggesting that HER2 and PD‐L1 play a vital role in guiding systemic treatment for patients with GC. This study aimed to depict the genomic and immune landscapes of Chinese patients with GC and investigate their correlations with HER2 amplification and PD‐L1 expression.

**Patients and Methods:**

Next‐generation targeted sequencing and PD‐L1 immunohistochemistry were performed on tumor samples from 735 patients with pathologically diagnosed GC. The genomic and immune landscapes and their correlations with HER2 amplification and PD‐L1 expression were analyzed.

**Results:**

The most commonly mutated genes in Chinese GC were *TP53* (64%), *CDH1* (20%), *ARID1A* (18%), *HMCN1* (15%), *KMT2D* (11%), and *PIK3CA* (11%). Seventy‐six (10%) patients were HER2 amplification, and 291 (40%) had positive PD‐L1 expression. Classifying the total population based on HER2 amplification and PD‐L1 expression level, 735 patients were divided into four subgroups: HER2+/PD‐L1+ (4.5%), HER2+/PD‐L1− (5.9%), HER2−/PD‐L1+ (35.1%), and HER2−/PD‐L1− (54.5%). The HER2+/PD‐L1− and HER2+/PD‐L1+ subgroups exhibited dramatically higher rate of *TP53* mutations, *CCNE1* and *VEGF* amplifications. The HER2+/PD‐L1− subgroup also had a markedly higher rate of *MYC* amplification and *KRAS* mutations. The HER2−/PD‐L1+ subgroup had significantly higher rate of *PIK3CA* mutations. HER2+/PD‐L1− subgroup had the highest TMB level and HER2−/PD‐L1+ subgroup had the highest proportion of patients with microsatellite instability‐high than other subgroups. Furthermore, we observed that different HER2 amplification levels had distinct impacts on the correlations between PD‐L1 expression and therapeutic genomic alterations, but no impact on the prognosis.

**Conclusion:**

The combination of HER2 amplification and PD‐L1 expression in Chinese patients with GC could stratify the total populations into several subgroups with distinctive genomic and immune landscapes, which should be considered when making personalized treatment decisions.

## Graphical abstract

The current study reported that combination of HER2 amplification and PD‐L1 expression in Chinese patients with gastric cancer could stratify the total populations into several subgroups with distinctive genomic and immune landscapes, which could be sensitive to different therapeutic regimens, indicating that both HER2 status and PD‐L1 expression level should be considered when making personalized treatment decisions for patients with advanced gastric cancer.

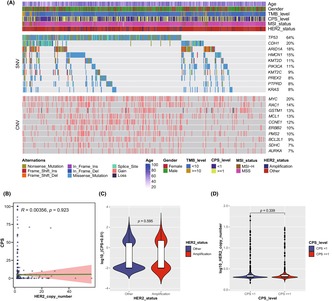

## 1 INTRODUCTION

Gastric cancer (GC) is the fifth most frequent cancer worldwide and the third leading cause of cancer‐related death, with dismal overall survival (OS). Over the past decade, molecular targeted therapies have optimized treatment options and markedly improved the OS of advanced GC. As an important subtype, GCs with human epidermal growth factor receptor 2 (HER2) amplification were identified in approximately 12% of gastric or gastro‐esophageal junction (G/GEJ) cases in China.^1^, ^2^, ^3^ In ToGA trial, trastuzumab (anti‐HER2 antibody) plus chemotherapy have shown the significantly OS benefit and become the standard first‐line treatment for HER2‐positive advanced G/GEJ cancers.^4^ After that, several randomized phase III trials in patients with HER2‐positive G/GEJ cancers also demonstrated the efficacy of HER2‐targeted therapies, including the TRIO‐013/LOGiC (lapatinib in combination with oxaliplatin and capecitabine as first‐line setting),^5^ the JACOB (trastuzumab plus pertuzumab and fluoropyrimidine and cisplatin as first‐line setting),^6^ the TyTAN (paclitaxel plus lapatinib as second‐line therapy)^7^ and the GATSBY (T‐DM1 as second‐line therapy) trials.^8^ These findings together indicate that HER2 is one of the significant therapeutic targets for patients with advanced GC.

Recently, immune checkpoint inhibitors targeting PD‐1 and PD‐L1 has emerged as a promising therapeutic regimen for patients with advanced GC. According to the newest National Comprehensive Cancer Network guidelines for advanced GC, nivolumab plus chemotherapy has been approved as the first‐line setting for patients with HER2‐negative advanced GC based on findings of CheckMate‐649 trial, particularly those with positive PD‐L1 expression (combined positive score [CPS] ≥5).^9^, ^10^ In addition, pembrolizumab monotherapy has been approved as second‐line or subsequent therapy for mismatch repair‐deficient (dMMR)/microsatellite instability high (MSI‐H) or high tumor mutation burden (≥10 mutations/megabase) tumors based on the findings of Keynote‐016 and Keynote‐158 studies.^11^, ^12^, ^13^, ^14^ More recently, the Keynote‐811 trial reported that pembrolizumab in combination with trastuzumab and chemotherapy as first‐line therapy significantly improved the efficacy than trastuzumab plus chemotherapy for patients with HER2 overexpression‐positive advanced GC. Moreover, in patients with PD‐L1 CPS ≥1, this triple treatment regimen showed a dramatically better objective response rate than those with negative PD‐L1 expression,^15^ suggesting that PD‐L1 is also the vital therapeutic target for patients with advanced or metastatic GC.

Given the importance of HER2 amplification and PD‐L1 expression in GC, this study aimed to depict the genomic and immune landscapes of GC patients and investigate their correlations with HER2 amplification and PD‐L1 expression. The impacts of HER2 amplification and PD‐L1 expression on prognosis were also explored in patients with surgically resected GC. These results revealed that patients with different HER2 amplification and PD‐L1 expression levels displayed distinctive genomic and immune profiles, together with different therapeutic genomic alterations, which should be considered when making personalized treatment decisions.

## 2 MATERIALS AND METHODS

### 2.1 Patients' selection and samples collection

A total of 735 patients with pathologically diagnosed GC were identified from Ruijin Hospital. Detailed features on clinical characteristics, such as sex, age, and pathologic stage, were collected. Age was recorded at initial diagnosis. We collected the data from electronic medical records via using the same requirements for clinical data on patient's follow‐up under treatment, including response to different treatments and clinical outcomes. Pretreatment fresh or archival formalin‐fixed paraffin‐embedded (FFPE) pretreatment tissue samples were collected before any systemic treatments. Fresh biopsy tissue samples were snap‐frozen in liquid nitrogen within half an hour. Baseline blood samples (8–10 mL) were collected in ethylene diamine tetraacetic acid‐coated tubes (BD Biosciences) and centrifuged at 1800*g* for 10 min within 2 h of collection to separate white blood cells. This study protocol was approved by the ethics committee and institutional review board of our center.

### 2.2 DNA extraction and next‐generation targeted sequencing

Genomic DNA was isolated from the tissue samples and matched peripheral blood lymphocytes by using the black PREP FFPE DNA Kit (Analytik Jena) and Tiangen Whole Blood DNA Kits (Tiangen, Beijing, PRC) according to the manufacturer's instructions. Then, genomic DNA was sheared into 150–200 bp fragments for sequencing with a Covaris M220 Focused‐ultrasonicator (Covaris) after quantified by a Qubit dsDNA HS Assay kit (Life Technologies). Fragmented DNA libraries were constructed using a KAPA HTP Library Preparation Kit (KAPA Biosystems, Massachusetts) according to the manufacturer's instructions. A custom capture panel (Genecast, Beijing, China) with 414 major tumor‐associated genes was applied to capture the DNA libraries. The captured DNA fragments were subjected to Novaseq 6000 processing for paired‐end sequencing.

### 2.3 Single nucleotide variants (SNVs) calling

The raw data with high‐quality reads were aligned to the human reference genome (Hg19, NCBI Build 37.5) using the Burrows‐Wheeler Aligner (BWA).^16^ Then, the Picard Toolkit and Genome Analysis ToolKit^17^ were utilized for making duplicates and realignment, respectively. After that, VarDict (version 1.5.1)^18^ was performed to call somatic SNVs while FreeBayes was performed to merge compound heterozygous mutations,^19^ and then ANNOVAR was applied to annotate the mutations.^20^ Paired genomic DNA samples were used as a control to distinguish somatic mutations from germline variations. The calling results were then filtered with custom. More stringent criteria were as follows: (a) mutant allele support reads ≥5; (b) not located in intergenic regions or intronic regions and not synonymous SNVs; (c) mutant allele frequency ≥5%; (d) allele frequency ≤ 0.2% in the ExAC database^21^ and Genome Aggregation Database.^22^

### 2.4 Copy number variation (CNV) calling

All blood samples obtained from patients were used to construct a copy number baseline for negative control and the CNVs from tissue samples were called for each patient using a CNVkit, V0.9.2. The thresholds of copy numbers (CNs) ≥3 and ≤1.2 were employed to categorize altered regions into CN gains (amplifications) and losses (deletions). Of note, the thresholds of CNs ≥3 were employed to categorize HER2 (*ERBB2*) amplification. Copy Number Variation burden was calculated as the number of copy number variant genes/per megabase for each patient.

### 2.5 TMB calculation

Only the regions with sequencing depth ≥100× after deduplication were utilized for TMB calculation. The TMB was defined as the number of somatic, base substitutions, coding, and inDel mutations per megabases of the examined genome. Germline alterations in the Single Nucleotide Polymorphism database or occurring with two or more counts in the ExAC database were not counted.

### 2.6 PD‐L1 staining

PD‐L1 expression evaluation was conducted according to the instructions of the PD‐L1 IHC 22C3 pharmDx kit (Agilent Technologies). CPS ≥1 was used as the cutoff to define positive PD‐L1 expression.

### 2.7 Identification of therapeutic genomic alterations by OncoKB

Therapeutic genomic alterations were defined by OncoKB (https://www.oncokb.org/) and classified into six therapeutic levels (Level 1: FDA‐approved drugs; Level 2: standard of care; Level 3: clinical evidence; Level 4: biological evidence; Level R1 and R2: two resistant levels). Individual mutational events were annotated by the level of evidence that supported the application of a certain drug.^23^

### 2.8 Statistical analyses

Clinicopathologic characteristics were summarized by number and percentages. Chi‐squared test or Fisher's exact test when needed was leveraged to compare the categorical variables. The continuous variables were analyzed by ANOVA and/or Tukey's multiple comparison tests. The Kaplan–Meier curves were used to estimate the median survival time of OS. Between‐group comparisons in OS were assessed using a stratified log‐rank test. The uni‐ and multivariate survival analyses were conducted using the Cox proportional hazards model and the hazard ratios (HRs) and corresponding 95% CIs were calculated and recorded. OS was defined as the interval from the date of the initial diagnosis to death from any reasons or was censored at the last follow‐up date. Last follow‐up was September 1, 2023. All statistical analyses were performed in the R software (https://www.r‐project.org/, version 4.0.5) and GraphPad Prism 8.0. Two‐sided *p* < 0.05 was considered statistically significant.

## 3 RESULTS

### 3.1 Baseline characteristics of included patients

The baseline parameters of 735 GC patients were summarized in Table 1. Briefly, the median age was 62 years (ranging from 52 to 69 years) and 62.7% were male patients. Of all patients, 443 had stage IV disease, 222 had stage III disease, and 70 had stage I/II disease. 10.3% patients had HER2 amplification. 39.6% of them had positive PD‐L1 expression (CPS ≥1). 5.6% of them had MSI‐H.

**TABLE 1:** Clinical characteristics of patients enrolled in this study.

Clinical characteristics	No. of patients
Total	735
Age (median [IQR])	62 [52, 69]
Gender (%)*	
Female	242 (32.9)
Male	493 (67.1)
Stage (%)*	
I	14 (1.9)
II	56 (7.6)
III	222 (30.2)
IV	443 (60.3)
MSI status (%)*	
MSS	690 (93.9)
MSI‐H	41 (5.6)
Unknown	4 (0.5)
HER2 status (%)*	
Amplification	76 (10.3)
Other	659 (89.7)
PD‐L1 status (%)*	
CPS≥1	291 (39.6)
CPS <1	444 (60.4)

Abbreviations: CPS, combined positive score; MSI, microsatellite instability; HER2, human epidermal growth factor receptor 2.

* Percentage indicates the proportion of patients with a specific clinical, pathologic, or molecular characteristic among all patients.

### 3.2 Different gene alteration patterns between HER2+ and HER2− subgroups

**FIGURE 1:**
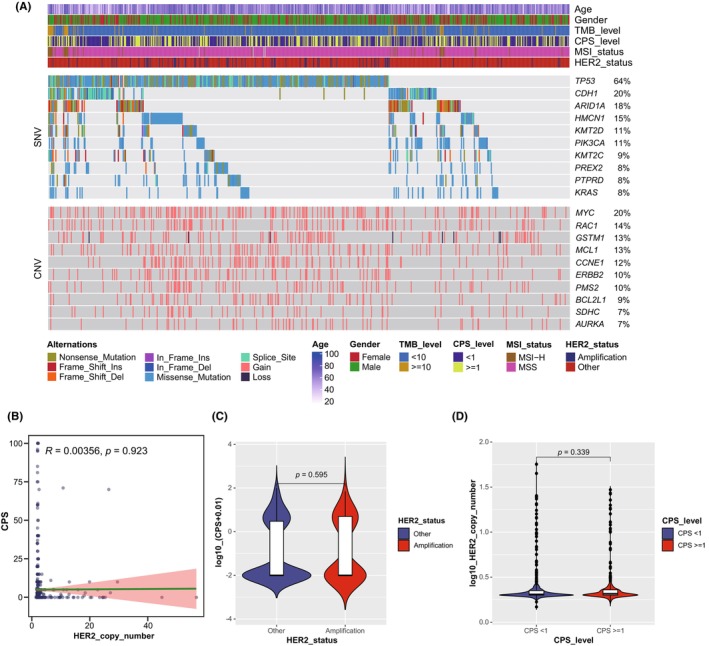
Genomic landscape of Chinese patients with GC. (A) The genomic landscape of 735 patients with GC. The top 10 altered genes with SNVs and CNVs were listed from high to low according to the frequencies plotted on the right panel. The top annotation showed the clinical characteristics of each patient. (B) Correlation of HER2 copy number and CPS. (C) Difference of CPS in different HER2 status. (D) Difference of *HER2* copy number in different PD‐L1 expression level. CNV, copy number variation; CPS, combined positive score; MSI, microsatellite instability; MSS, microsatellite stable; SNV, single nucleotide variant; TMB, tumor mutation burden.

First, to identify the significant genomic alterations, we explored the SNV and CNV profiles in all patients. The most commonly mutated genes were *TP53* (64%), followed by *CDH1* (20%), *ARID1A* (18%), *HMCN1* (15%), *KMT2D* (11%), and *PIK3CA* (11%). The results were coincident with previously reports that alterations in some genes, such as *TP53*,^24^ *KRAS*,^25^ *PIK3CA*, *ERBB2*, *ERBB4*, and *NF1* ^26^ were commonly mutated and may be associated with drug resistance. Copy Number Variation analysis showed that the somatic CNV gain was the dominant type in Chinese GC patients and the top somatic CNV genes were *MYC* (20%), *RAC1* (14%), *GSTM1* (13%), *MCL1* (13%), and *CCNE1* (12%) (Figure 1A).

To gain insights into the genomic landscape of tumors with HER2 amplification, we stratified all patients into HER2+ (HER2 amplification) and HER2− subgroups (HER2 other). The baseline characteristics of two groups were listed in Table S1. SNV analysis showed that *TP53* mutational rate was prominently higher in HER2+ subgroup compared to HER2− subgroup (87% vs. 61%, *p* < 0.01), followed by *HMCN1* (22% vs. 14%, *p* < 0.01) and *KMT2C* (11% vs. 9%, *p* < 0.05). On the contrary, mutational rates of *CDH1* (8% vs. 21%, *p* < 0.01), *ARIDI1* (11% vs. 18%, *p* < 0.01), and *PIK3CA* (8% vs. 11%, *p* < 0.05) were lower in HER2+ subgroup (Figure S1A). The significantly altered SNVs in HER2+ subgroup were shown in Figure S1B. Copy Number Variation analysis showed that *ERBB2* (100% vs. 0%, *p* < 0.001), *MYC* (41% vs. 17%, *p* < 0.01), *CCNE1* (32% vs. 10%, *p* < 0.01), *RAC1* (25% vs. 12%, *p* < 0.01), and *MCL1* (24% vs. 11%, *p* < 0.01) were especially enriched in HER2+ subgroup than in HER2− subgroup. Particularly, several genes including *ERBB2, CDK12, RARA*, and *BRCA*, showed significant copy number gains in HER2+ subgroup (Figure S1C), whereas some genes such as *APC, RASA1, FAS*, and *ERG*, showed obvious copy number loss (Figure S1D).

**FIGURE 2:**
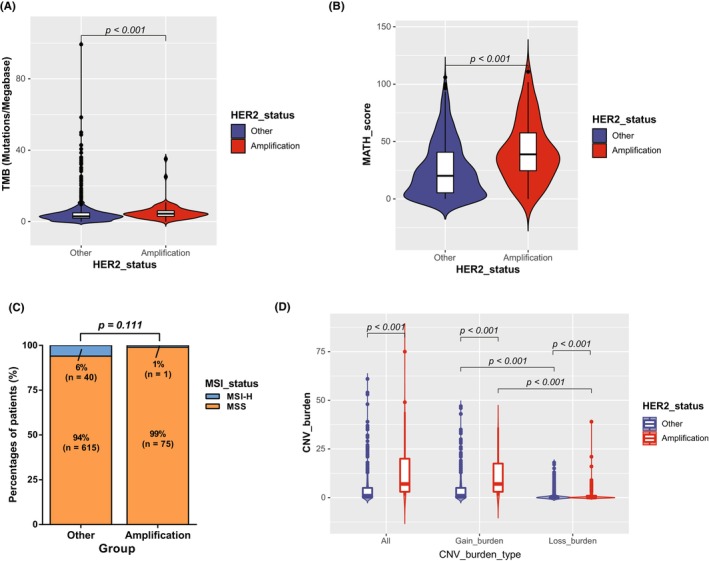
Differential gene alteration patterns in HER2+ versus HER2− subgroups. (A) Difference of TMB in different HER2 status. (B) Difference of MATH in different HER2 status. (C) Distribution of MSI status in different HER2 status. (D) CNV type and burden in different HER2 status. CNV, Copy number variation; CPS, combined positive score; MATH, mutant‐allele tumor heterogeneity; MSI, microsatellite instability; MSS, microsatellite stable; TMB, tumor mutation burden.

HER2 amplification, PDL1 CPS and MSI status are the promising predictive biomarkers for patients with GC received systemic antitumor treatments. To evaluate the associations between HER2 expression and immunotherapy‐related features, we compared the PD‐L1 CPS, TMB level,^27^, ^28^ MSI status,^29^ and mutant‐allele tumor heterogeneity (MATH)^30^ between HER2+ and HER2− subgroups. The results showed that PD‐L1 CPS was not associated with HER2 amplification levels (*R* = 0.004, *p* = 0.923, Figure 1B). Concordantly, no significant difference of PD‐L1 CPS between HER2+ and HER2− subgroups was observed (*p* = 0.595, Figure 1C). HER2 amplification levels were analogous in different PD‐L1 CPS subgroups (*p* = 0.339, Figure 1D), indicating independent predictive value of HER2 amplification and PD‐L1 CPS in GC. However, HER2+ subgroup had significantly higher TMB level (Figure 2A), MATH score (Figure 2B) but comparable MSI status when compared with HER2− subgroup (Figure 2C). Additionally, HER2+ subgroup had higher CNV burden, especially copy number gain burden than HER2− subgroup (Figure 2D).

### 3.3 Genomic and immune landscapes in patients with distinct HER2 amplification and PD‐L1 expression level

**FIGURE 3:**
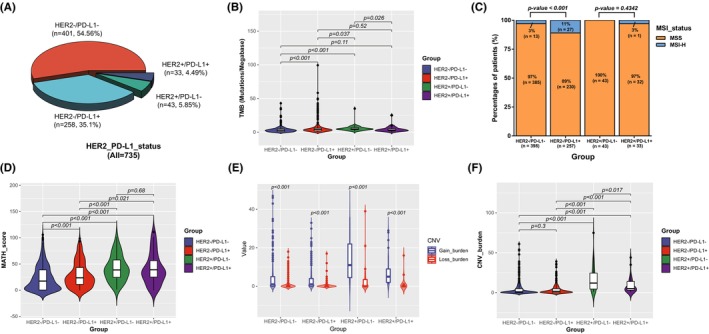
Classification of all patients into four subgroups using HER2 amplification and PD‐L1 expression. (A) Distribution of different HER2 and PD‐L1 status. HER2+/−: HER2 amplification or other, PD‐L1+/−: CPS ≥1 or <1. (B) Difference of TMB in different groups. (C) Distribution of MSI status in different groups. (D) Difference of MATH in different groups. (E) Difference of CNV type and value in different groups. (F) Difference of CNV burden in different groups. CNV, Copy number variation; CPS, combined positive score; MATH, mutant‐allele tumor heterogeneity; MSI, microsatellite instability; TMB, tumor mutation burden.

According to HER2 amplification and PD‐L1 expression, GC patients were stratified into four subgroups for further analysis (Figure 3A), including HER2−/PD‐L1− (54.56% [401/735], HER2 other and PD‐L1 CPS <1), HER2−/PD‐L1+ (35.1% [258/735], HER2 other and PD‐L1 CPS ≥1), HER2+/PD‐L1− (5.85% [43/735], HER2 amplification and PD‐L1 CPS <1) and HER2+/PD‐L1+ (4.49% [33/735], HER2 amplification and PD‐L1 CPS ≥1). Totally, HER2+/PD‐L1− subgroup had the highest median TMB level (Figure 3B) and HER2−/PD‐L1+ subgroup had the highest proportion of patients with MSI‐H status than other subgroups (Figure 3C). Among HER2− patients, PD‐L1+ subgroup has higher TMB level, MATH score and proportion of MSI‐H when compared with PD‐L1− subgroup. Whereas, there was no significant difference in the proportion of MSI‐H and MATH score between PD‐L1+ and PD‐L1− subgroups among HER2+ patients (Figure 3B–D). Additionally, all four subgroups had higher copy number gain than copy number loss burden (Figure 3E) and HER2+/PD‐L1− subgroup had the highest CNV burden than other subgroups (Figure 3F).

**FIGURE 4:**
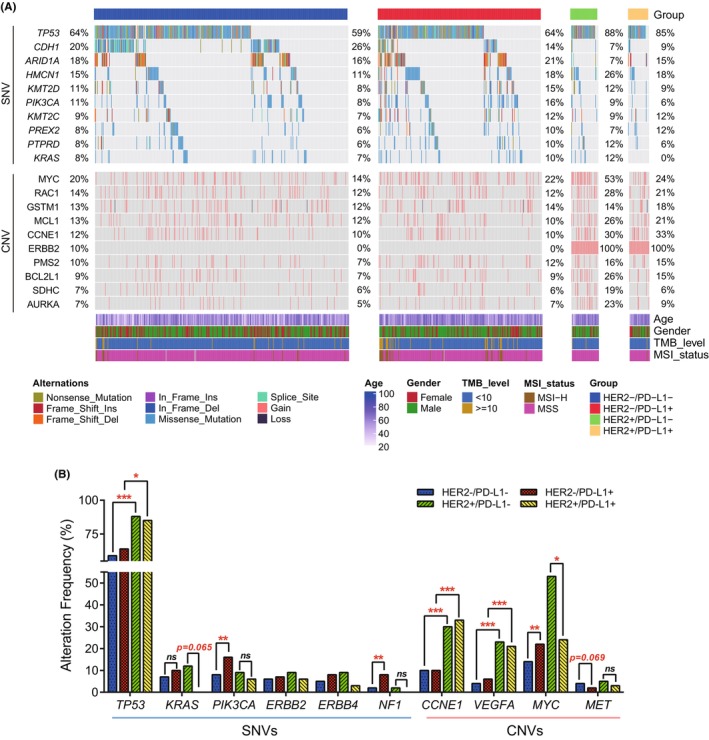
Genomic landscape in patients with different HER2 and PD‐L1 expression. (A) The mutational profiles comparing HER2−/PD‐L1−, HER2−/PD‐L1+, HER2+/PD‐L1−, and HER2+/PD‐L1+. The top 10 frequent SNVs and CNVs in the study cohort were shown with mutation frequencies in each subgroup indicated. (B) Percentages of SNVs and CNVs that related to drug target or resistance among the subgroups. *, **, and *** represented the *p*‐value <0.05, *p*‐value <0.01, and *p*‐value <0.001, respectively. CNV, copy number variation; CPS, combined positive score; MSI, microsatellite instability; MSS, microsatellite stable; SNV, single nucleotide variant; TMB, tumor mutation burden.

To gain a further insight into genomic alterations associated with different HER2 and PD‐L1 expression status, we compared SNV and CNV profiles among four subgroups (Figure 4A). As shown in Figure 4B, *TP53* mutation frequency was prominently higher in the HER2+/PD‐L1− (88%) and HER2+/PD‐L1+ (85%) subgroups than the HER2−/PD‐L1+ (64%) and HER2−/PD‐L1− (59%) subgroups. However, neither the variant types nor the mutated exons of *TP53* significantly differed among the four subgroups (Figure S2A). Moreover, *KRAS* mutations were detected in 12% of HER2+/PD‐L1− patients, but none in HER2+/PD‐L1+ subgroup. Mutations in *PIKC3A* and *NF1* were significantly enriched in HER2−/PD‐L1+ subgroup compared to other subgroups. Additionally, there was no significant difference in the mutant frequency of *ERBB2* and *ERBB4* among four subgroups (Figure 4B). The top frequently altered genes with various copy number were also shown in Figure 4A. The results indicated that the CNVs frequency of *CCNE1* and *VEGFA* were much higher in the HER2+/PD‐L1+ group than in the HER2−/PD‐L1+ and HER2−/PD‐L1− groups, but did not differ from the HER2+/PD‐L1− group. The CNV frequency of *MYC* was markedly higher in HER2+/PD‐L1− subgroup than the HER2+/PD‐L1+ subgroup, which was consistent with previously reported transcriptional regulation of *MYC* on PD‐L1,^31^ however, the regulation was HER2 amplification dependent and not applicable in HER2− subgroups (Figure 4B). Particularly, we found a high concurrent amplification of *CDK12* in HER2+ GC patients (Figure S2B), which amplification was reported to sensitize/re‐sensitize HER2+ breast cancer to lapatinib.^32^ Further investigation by spearman correlation analysis revealed that the copy numbers of *CDK12* were positively correlated with *HER2* copy numbers (R = 0.381, *p* < 0.001) (Figure S2C). Among the four GC subgroups, *CDK12* copy number amplifications were dominantly enriched in HER2+ subgroups, especially in HER2+/PDL1‐ subgroup (Figure S2D), which was coincident with the distribution of *CDK12* amplification (Figure S2E).

### 3.4 Comparison of distinct enriched signaling pathways among four subgroups

**FIGURE 5:**
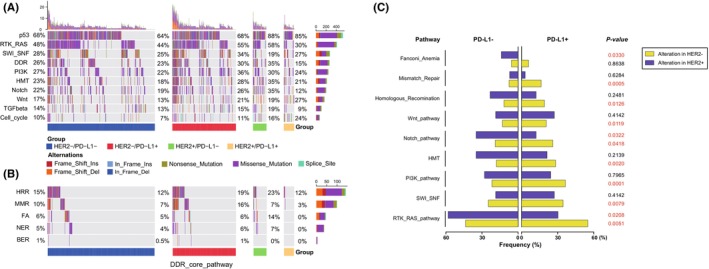
Comparison of distinct enriched signaling pathways among subgroups. (A and B) The landscape of the top 10 cancer‐associated classical pathways and DDR core pathways in different subgroups. BER, base excision repair; DDR, DNA damage repair; FA, fanconi anemia; HMT, histone methyltransferase; HRR, homologous recombination repair; MMR, mismatch repair; NER, nucleotide excision repair; (C) Mutational frequencies of pathways associated with HER2 and PD‐L1 statuses.

Having noticed the distinct mutational landscape among patients with different HER2 and PD‐L1 expression status, we then investigate the enriched signaling pathways among different subgroups. The results showed that the p53 pathway (68%) had the highest frequency, followed by the RTK‐RAS pathway (48%), SWI/SNF (28%), and the DNA damage repair (DDR) pathway (26%) (Figure 5A). The p53 pathway was significantly enriched in the HER2+ subgroups. Percentage of cell cycle pathway alterations was highest in the HER2+/PD‐L1+ subgroup, which had relatively lower frequency in the RTK‐RAS, DDR, Notch, and TGF‐β pathways.

Additionally, we analyzed the alterations in the DDR core pathways, including mismatch repair (MMR), base excision repair (BER), homologous recombination repair (HRR), nucleotide excision repair (NER), non‐homologous end‐joining (NHEJ), Fanconi anemia (FA), checkpoint factors (CPF), and translesion DNA synthesis.^33^ As presented in Figure 5B, HRR had the highest frequency (15%), followed by MMR (10%), FA (6%), NER (5%), and BER (1%). HRR pathway alterations were reported to be more sensitive to poly ADP‐ribose polymerase inhibitors. Its alteration rate was obviously higher in the HER2−/PD‐L1+ and HER2+/PD‐L1− subgroups.

Also, we found that positive PD‐L1 expression was associated with the enrichment of RTK‐RAS, SWI/SNF, PI3K, histone methyltransferase (HMT), Notch, Wnt, HRR, and MMR pathways in HER2− patients, which was not observed in HER2+ patients. Moreover, for some pathways including the RTK‐RAS and Notch pathways, the correlation between PD‐L1 and pathway enrichments was opposite in HER2+ versus HER2− groups (Figure 5C).

### 3.5 Therapeutic genomic alterations in Chinese GC

**FIGURE 6:**
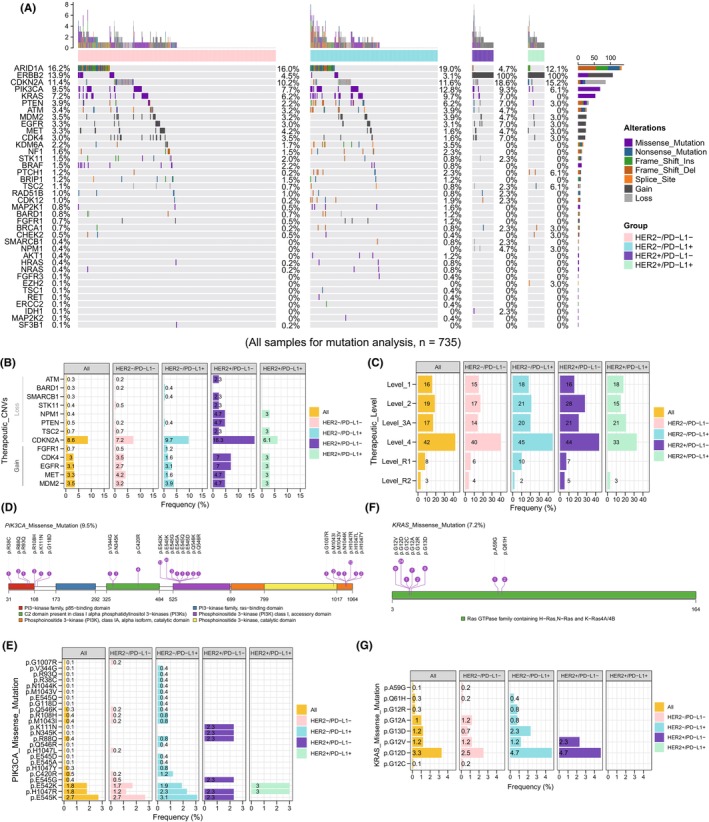
Therapeutic genomic alterations in Chinese GC cohort. (A) The landscape of OncoKB‐defined therapeutic genomic alterations. Distribution of therapeutic CNVs (B) and levels (C) in different subgroups. Locations of therapeutic SNVs and distribution of common mutation hotpots in *PIK3CA* (D–E) and *KRAS* (F–G) in different subgroups. CNV, copy number variation; SNV, single nucleotide variant.

To further elucidate the value of somatic alterations in GC, we compared the genomic alterations identified in this study with those in the OncoKB database. In 417 (56.7%) patients of this study, at least one OncoKB‐defined therapeutic genomic alteration was identified in this or another indication that may benefit from targeted therapy. A total of 774 oncogenic or likely oncogenic therapeutic genomic alterations in 38 genes identified in this study were recorded for pan‐cancer in the OncoKB database. Therapeutic genomic alterations included 514 mutations (SNVs and indels) and 260 CNVs. The top five genes with genomic alterations were *ARID1A* (16.2%), *ERBB2* (13.9%), *CDKN2A* (11.4%), *PIK3CA* (9.5%), and *KRAS* (7.2%) (Figure 6A). In addition, druggable genes with frequent alterations in CNVs were mostly found in *CDKN2A* (8.6%), *MDM2* (3.5%), *MET* (3.3%), *EGFR* (3.1%), and *CDK4* (3%) in the Chinese GC cohort (Figure 6B). And therapeutic CNVs occurred more frequently in the HER2+/PD‐L1− subgroup. Moreover, without considering HER2 amplification, level 1, level 2, level 3A, and level 4 therapeutic genomic alterations accounted for 16%, 19%, 17%, and 42% in this GC cohort, respectively (Figure 6C). The distribution of patients with different therapeutic levels in altered genes was also displayed in Figure S3.

For *PIK3CA* missense mutations, the therapeutic hotspots were found in the p85‐binding domain (exon 2, p.R38C, p.R88Q, p.R93Q, p.R108H, p.K111N, and p.G118D), the C2 domain (exon 5, p.N345K, exon 8, p.V344G, p.C420R, and p.E453K), the helical (exon 10, p.E542K, p.E545A/D/G/K/Q, and p.Q546K/R) and the catalytic domain (exon 21, p.G1007R, p.M1043I/V, p.N1044K, and p.1047L/R/Y) (Figure 6D). Among the mutant sites in *PIK3CA*, amino acids change of p.E545K/ p.E542K/ p.H1047R occurred most frequently. Mutant rate of p.H1047R was a bit lower in HER2−/PD‐L1− group. *PIK3CA* p.E545K and p.E542K were not found in HER2+/PD‐L1+ and HER2+/PD‐L1− subgroups, respectively (Figure 6E).

In *KRAS* missense mutations, the therapeutic mutational hotspots were found in exon 2 (p.G12A, p.G12R, p.G12V, p.G12C, p.G12D, and p.G13D) and exon 3 (p.A59G and p.Q61R) (Figure 6F). In addition, *KRAS* p.G12D exhibited extremely high mutation rate in both HER2−/PD‐L1+ (4.7%) and HER2+/PD‐L1− (4.7%) subgroups, while *KRAS* p.G13D was more frequently observed in the HER2−/PD‐L1+ subgroup (Figure 6G). Intriguingly, no case in the HER2+/PD‐L1+ subgroup had therapeutic *KRAS* mutations.

### 3.6 The associations between prognosis and HER2 status and PD‐L1 expression in early‐stage GC

**FIGURE 7:**
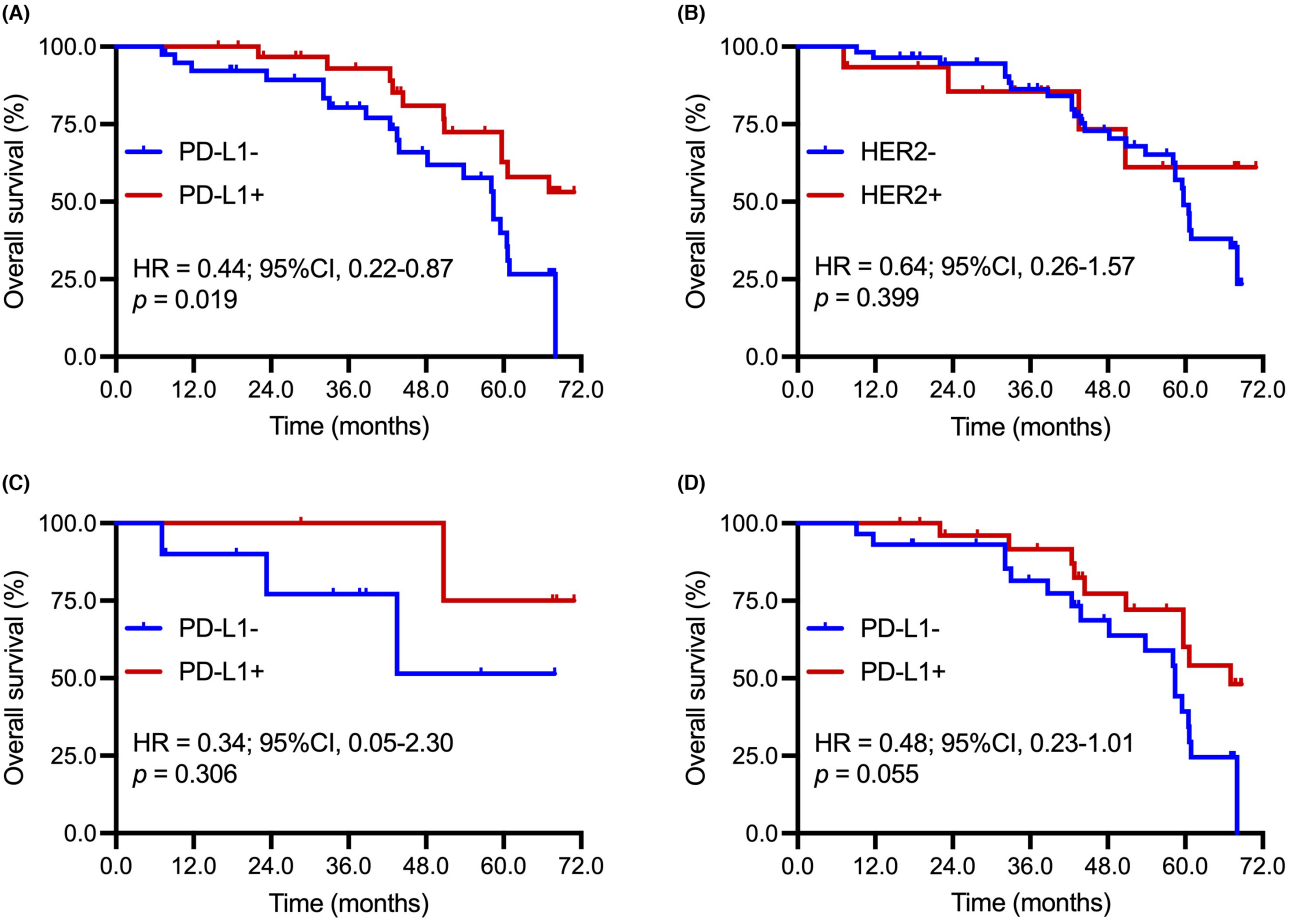
Associations between prognosis and HER2 status and PD‐L1 expression in early‐stage GC. (A) Comparison of OS between patients with positive and negative PD‐L1 expression in patients with early‐stage gastric cancers. (B) Comparison of OS between patients with and without HER2 amplification in patients with early‐stage gastric cancers. (C) Comparison of OS between patients with positive and negative PD‐L1 expression in patients with HER2 amplification. (D) Comparison of OS between patients with positive and negative PD‐L1 expression in patients without HER2 amplification.

To further investigate the impacts of different HER2 status and PD‐L1 expression on the overall prognosis of patients with surgically resected GC, we collected survival data of patients with early‐stage GC received surgery. The results showed that positive PD‐L1 expression was associated with a better OS after surgical resection in patients with early‐stage gastric cancers (HR = 0.44, 95%CI: 0.22–0.87; *p* = 0.019; Figure 7A), but HER2 amplification was not correlated with prognosis in these population (HR = 0.64, 95%CI: 0.26–1.57; *p* = 0.399; Figure 7B). Considering different HER2 amplification levels had distinct impacts on the correlations between PD‐L1 expression and therapeutic genomic alterations, we then analyzed the prognostic value of PD‐L1 expression in patients with or without HER2 amplification. It seemed that positive PD‐L1 expression was associated with favorable prognosis irrespective of HER2 status (Figure 7C,D).

## 4 DISCUSSION

Anti‐PD1/PD‐L1 antibody plus HER2 antibody and chemotherapy have become the new first‐line therapy for HER2 overexpression‐positive advanced GC, suggesting that HER2 and PD‐L1 play a significant role in guiding personalized treatment for patients with GC. Nevertheless, not all of them can benefit from this combination treatments and identification of the potential benefit populations from anti‐PD1/PD‐L1 antibody plus trastuzumab and chemotherapy is urgently needed in current clinical practice. Considering the significant impact of genomic and immune profiles on therapeutic efficacy, the present study provides the largest cohort of Chinese patients with GC to depict the genomic and immune landscapes and their correlations with HER2 amplification and PD‐L1 expression.

HER2 amplification is one of the identified therapeutic targets in advanced GC. Several elegant phase III trials including ToGA, TRIO‐013/LOGiC, JACOB, and GATSBY have consistently demonstrated HER2‐targeted therapies could improve the efficacy and/or prognosis of HER2‐positive advanced GCs. In line with previous studies, the current study reported that 10.3% of patients were identified as HER2‐amplified GC.^1^, ^2^, ^3^ Genomic analysis revealed that HER2+ GC had obviously distinct mutational landscape when compared with HER2− GC. HER2+ subgroup also had significantly higher TMB level and MATH score but comparable PD‐L1 expression level and MSI status when compared with HER2− subgroup, indicating that GC patients with HER2 amplifications may be one of the potential benefit populations. Moreover, 4.5% of patients had both HER2 amplification and positive PD‐L1 expression in our cohort. They could be more suitable for receiving anti‐PD1/PD‐L1 antibody plus trastuzumab and chemotherapy. These findings together suggest that HER2 status could impact the genomic and immune profiles of GCs.

PD‐L1 expression level detection could be influenced by various factors including immunohistochemistry scoring methods, test antibodies, and cut‐off definitions, which vary across different studies and are difficult for directly cross comparison.^34^ Thus, additional biomarkers are urgently needed to identify the potential benefit candidates from anti‐PD‐1 therapy. Herein, we made a great effort to elucidate the genetic mutations correlated with PD‐L1 expression. Surprisingly, we found that TMB, MSI‐H, and MATH were positively correlated with high PD‐L1 expression in HER2− patients, which is in line with the observation in a larger cohort of GC patients.^35^ However, the HER2+ group did not show the same results. Similarly, high PD‐L1 expression was observed to be associated with mutations in RTK‐RAS, SWI/SNF, PI3K, HMT, Notch, Wnt, HRR and MMR pathways in HER2− patients, which was inconsistent with the findings in HER2+ patients. Furthermore, even for some pathways, including RTK‐RAS and Notch pathways, the correlation between PD‐L1 and pathway enrichments was opposite between HER2+ and HER2− patients, suggesting HER2 status would have impact on the signaling pathway enrichments in patients with different PD‐L1 expression. All these findings suggested that when we made the treatment decisions and searched the potential benefit candidates based on genomic features, we should also take the HER2 status and PD‐L1 expression level into consideration.

In addition, actionable aberrations interpreted by oncoKB were evaluated in all patients. Around 57% (417/735) of GC patients had at least one actionable alteration. This proportion was a bit higher than that in another Chinese GC cohort, probably due to the update of the oncoKB database and the larger cohort we explored.^36^ In the present study, most of the actionable genetic alterations were not specific. They could be widely observed throughout all subtypes. But the most frequent mutations in *BRAF*, *KDM6A*, and *NF1* were only observed in HER2− patients, indicating that the likelihood of targeting these genes in HER2+ patients was low. Previous studies reported that gastrointestinal cancers are characterized by *ARID1A* mutations,^37^ which were correlated with higher PD‐L1 expression level. In our cohort, we also observed the correlation between *ARID1A* mutation and PD‐L1 expression in patients with HER2 amplification. However, in HER2+/PD‐L1− subgroup, frequency of *ARID1A* mutation was lower than other groups.

For HER2−/PD‐L1− patients, potential targeted therapies remain undetermined. Among these patients, four therapeutic altered genes with frequency >5% were identified, including mutations in *ARID1A*, *PIK3CA* and *KRAS*, and *CDKN2A* loss. Similarly, a previous publication reported that the common therapeutic mutation hotspots enriched in *PIK3CA* p.E545K, p.H1047R, and p.E542K could help alpelisib plus fulvestrant achieve greater clinical activity.^38^ As previous publications shown, *CDKN2A* belongs to the cell cycle pathway, which inhibits the activity of CDK4/6.^39^ In our study, over 16% of patients have *CDKN2A* loss, indicating that these patients could potentially benefit from CDK4/6 inhibitors. In addition, drugs targeting *KRAS* G12C have been approved by FDA for *KRAS* G12C mutant advanced non‐small‐cell lung cancer.^40^ Despite its low frequency (0.2%), it still gives hope to GC patients who harbored *KRAS* G12C mutation. These results indicated that there were opportunities for targeted therapies in HER2−/PD‐L1− patients. The safety and efficacy of these above‐mentioned molecular targeted drugs warrant further investigation in HER2−/PD‐L1− patients.

Given the correlations between HER2 amplification and PD‐L1 expression, and genomic and immune landscapes, we also investigated the impacts of HER2 amplification and PD‐L1 expression on overall prognosis in patients with surgically resected GC. The present findings showed that positive PD‐L1 expression was associated with favorable prognosis after surgical resection in patients with early‐stage gastric cancers irrespective of HER2 status, while HER2 amplification was not correlated with prognosis in these populations. Consistently, Chen et al. analyzed the prognostic value of PD‐L1 expression in 147 gastric cancer patients with peritoneal metastasis and found that high PD‐L1 expression was the independent and significantly favorable prognostic factor.^41^ Shen performed a large‐scale multicenter study involving 1562 GC patients treated by R0 resection and reported that positive status of HER2 was not related to the survival in patients with GC among the Chinese population.^42^ Notably, Gao et al. involved 5622 consecutive stage II/III GC patients and observed that HER2 overexpression was independently associated with a lower 5‐year OS in stage II, but not in stage III GC.^43^ These findings together revealed that the impacts of HER2 amplification and PD‐L1 expression on prognosis in patients with GC remains controversial and should be re‐considered under different circumstances (e.g., clinical, pathological stage, genomic, transcriptomic, and immune landscapes).

This study had several limitations that should be acknowledged. First, the small sample size with retrospective nature will inevitably have selection bias. Therein, the findings should be cautiously interpreted. Future large‐scale prospective study is still warranted. Second, HER2 status was defined by targeted NGS sequencing, which could not completely replace the detection of IHC or FISH. Third, the low frequency of HER2 amplification limits the efficacy of statistical tests in HER2+ patients. Forth, we only performed the targeted sequencing on the included tumor samples. The single omics approach could only provide very limited information. Considering the transcriptomic and immune features of tumors with distinct PD‐L1 and HER2 expression level are significant to guide the treatment decision, multi‐omic data should be analyzed and added in the future investigations. Last but not least, clinical trials with a large number of patients with GC administrated with immunotherapy are needed to further confirm the predictive value of the genomic factors correlated with PD‐L1 expression.

In summary, combination of PD‐L1 expression and HER2 amplification in Chinese patients with GC could stratify the total populations into several subgroups with distinctive genomic and immune landscapes, which could be sensitive to different therapeutic regimens. The current findings indicate that both HER2 status and PD‐L1 expression level should be taken into consideration when making personalized treatment decisions for patients with advanced GC.

## AUTHOR CONTRIBUTIONS

**Xiaoqian Jing:** Data curation (lead); methodology (equal); project administration (lead); supervision (supporting); writing – original draft (supporting); writing – review and editing (supporting). **Zhiping Luo:** Data curation (equal); methodology (equal); project administration (equal); writing – original draft (supporting). **Jiayan Wu:** Data curation (equal); methodology (supporting). **Feng Ye:** Data curation (supporting); methodology (supporting). **Jianfang Li:** Data curation (supporting); methodology (supporting). **Zijia Song:** Data curation (supporting); methodology (supporting). **Yaqi Zhang:** Data curation (supporting); methodology (supporting). **MinMin Shi:** Data curation (supporting). **Huaibo Sun:** Data curation (supporting). **Yi Fang:** Data curation (lead); methodology (equal); project administration (supporting); writing – original draft (lead); writing – review and editing (supporting). **Yimei Jiang:** Formal analysis (supporting); investigation (supporting); methodology (supporting); project administration (lead); supervision (lead); writing – original draft (supporting); writing – review and editing (supporting). **Xiaopin Ji:** Conceptualization (lead); formal analysis (lead); funding acquisition (lead); methodology (lead); writing – original draft (lead); writing – review and editing (lead).

## FUNDING INFORMATION

This study was partly sponsored by National Natural Science Foundation of China (81902374), the Youth Development Program of Shanghai Tenth People's Hospital (04.03.19.140), and the Youth Development Program of Ruijin Hospital, Shanghai Jiao Tong University School of Medicine (KY20211514).

## CONFLICT OF INTEREST STATEMENT

Jiayan Wu and Huaibo Sun were employed by the Genecast Biotechnology Co., Ltd, Wuxi, China. Other authors declared no potential conflict of interest.

## ETHICS STATEMENT

This study protocol was approved by the Ethics Committee and Institutional Review Board of Ruijin Hospital (RJ‐2022‐214). Since this is a retrospective analysis, a waiver on the patient's written informed consent was granted by the Institutional Review Board of Ruijin Hospital.

## Supporting information


Appendix S1.

## Data Availability

The data that support the current findings of this study are available from the lead author upon reasonable request. All requests for raw data will be reviewed by the leading clinical site to check whether the request is subject to any intellectual property or confidentiality obligations. Source data are provided with this paper. A signed data access agreement with the sponsor is required before accessing shared data.
