# Supplementary material for: The genomic and immune landscapes of gastric cancer and their correlations with HER2 amplification and PD‐L1 expression
Source: Cancer Med. 2023 Dec 5;12(24):21905–19. doi: 10.1002/cam4.6765 (PMC10757096; doi:10.1002/cam4.6765)
Supplement: Supplementary file 1 — Appendix S1. [file CAM4-12-21905-s001.docx]

**Supplementary Materials**

**Supplementary Table 1.** Clinical characteristics of HER2 amplification versus HER2 other molecular subgroups of GC

| **Clinical characteristics** | **HER2_amplification**  **(*N* = 76)** | **HER2_other**  **(*N* = 659)** | ***p*-value** |
| --- | --- | --- | --- |
| Age (median [IQR]) | 65.00 [57.75, 71.00] | 61.00 [51.50, 68.00] | ***0.003*** |
| Gender (%) |  |  | 0.898 |
| Female | 24 (31.6) | 218 (33.1) |  |
| Male | 52 (68.4) | 441 (66.9) |  |
| Stage (%) |  |  | 0.637 |
| Ⅰ | 1 (1.3) | 13 (2.0) |  |
| Ⅱ | 5 (6.6) | 51 (7.7) |  |
| Ⅲ | 28 (36.8) | 194 (29.4) |  |
| Ⅳ | 42 (55.3) | 401 (60.8) |  |
| MSI_status (%) |  |  | 0.628 |
| MSS | 70 (92.1) | 620 (94.1) |  |
| MSI-H | 6 (7.9) | 35 (5.3) |  |
| unknown | 0 (0.0) | 4 (0.6) |  |
| PD-L1_status |  |  | 0.536 |
| CPS ≥ 1 | 33 (43.4) | 258 (39.2) |  |
| CPS < 1 | 43 (56.6) | 401 (60.8) |  |

HER2: human epidermal growth factor receptor 2; MSI: microsatellite instability; CPS: combined positive score. *Percentage indicates the proportion of patients with a specific clinical, pathologic, or molecular characteristic among all patients.


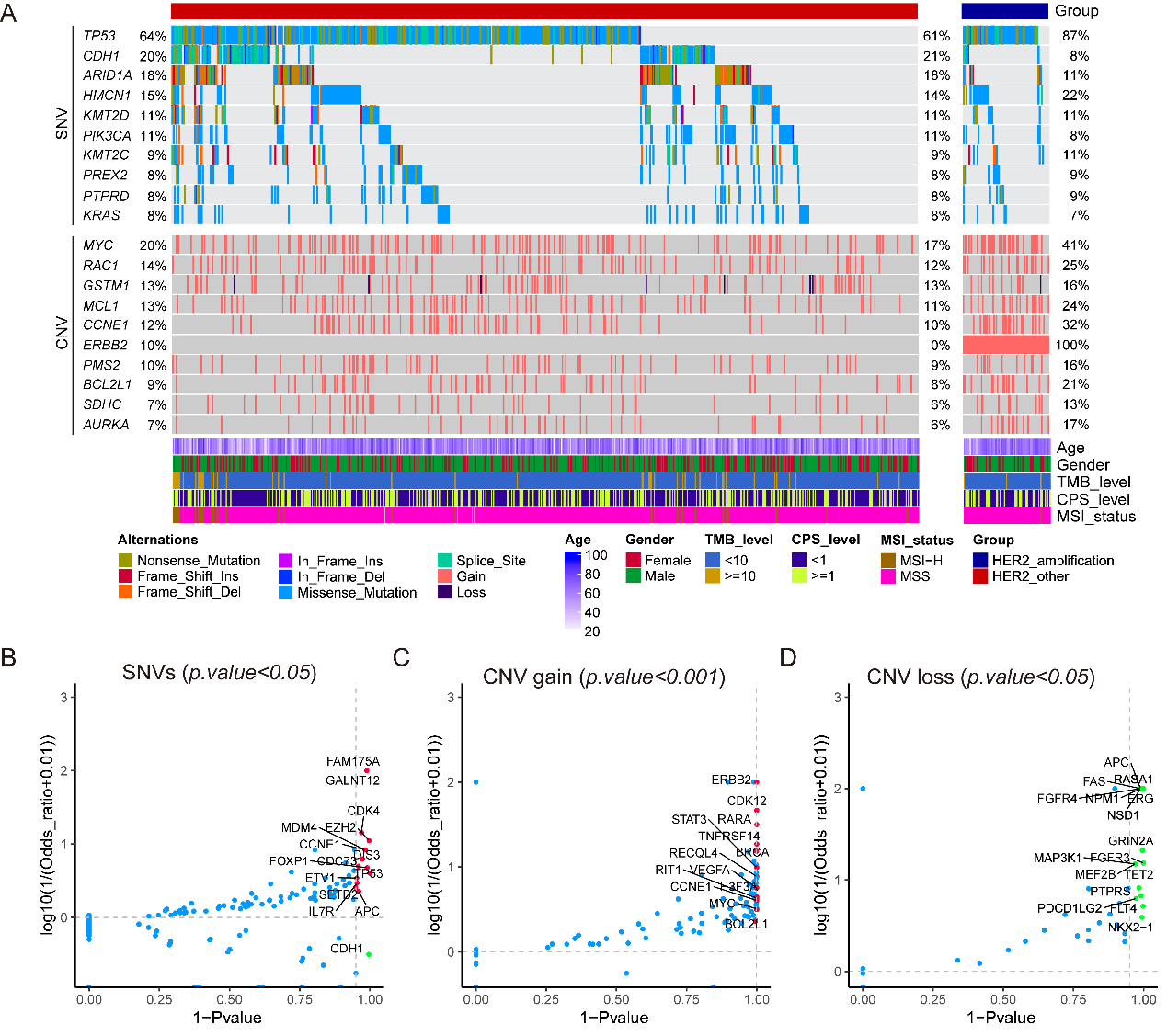


**Supplementary Fig. S1 Genetic alterations analysis in HER2 amplification and HER2 other patients**. (A) Mutational profiles comparing HER2 amplification and HER2 other. The top ten frequent SNVs and CNVs in the study cohort were shown with mutation frequencies in each subgroup indicated. Significantly altered genes of (B) SNVs, (C) CNV gains (amplification), and (D) CNV losses (deletions). A blue dot indicated a not significantly altered gene between subgroups. A red dot indicated a significantly altered gene and had a higher alteration frequency in HER2 amplification. A green dot indicated a significantly altered gene and had a higher alteration frequency in HER2 other. The thresholds of *p*-value for the SNVs, CNV gain, and CNV loss were < 0.05, < 0.001, and < 0.05, respectively.


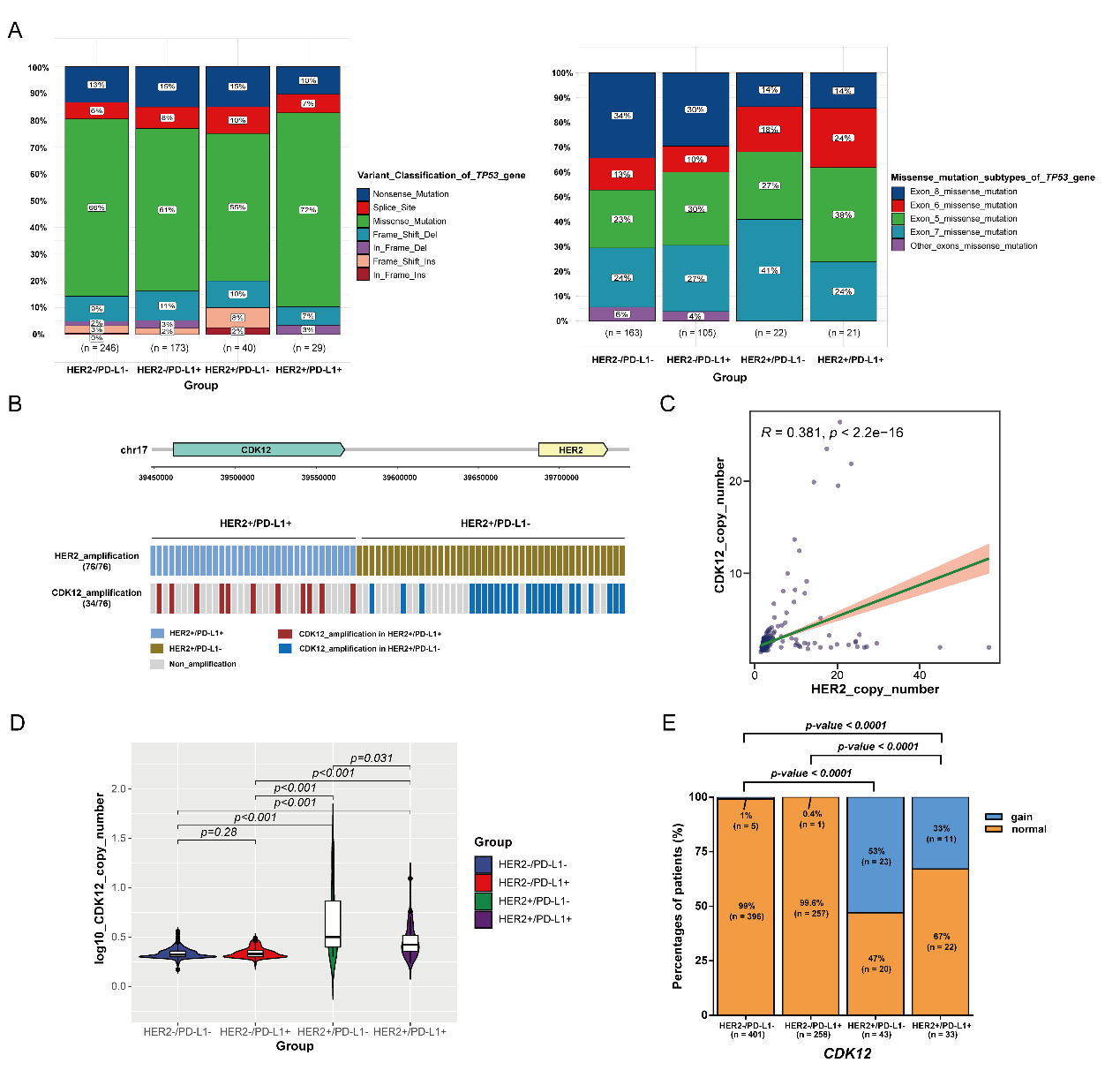


**Supplementary Fig. S2 Further analysis of *TP53* gene and *CDK12* co-amplified with *HER2***. (A) Distribution of variant classification and missense mutation subtypes of *TP53* gene in different groups. (B) Chromosome locations of *CDK12* and *HER2*. (C) Correlation of *CDK12* copy number with *HER2* copy number of 735 GC patients. (D) Between-group variation of *CDK12* copy number among the subgroups. (E) Distribution of *CDK12* amplification in different groups.


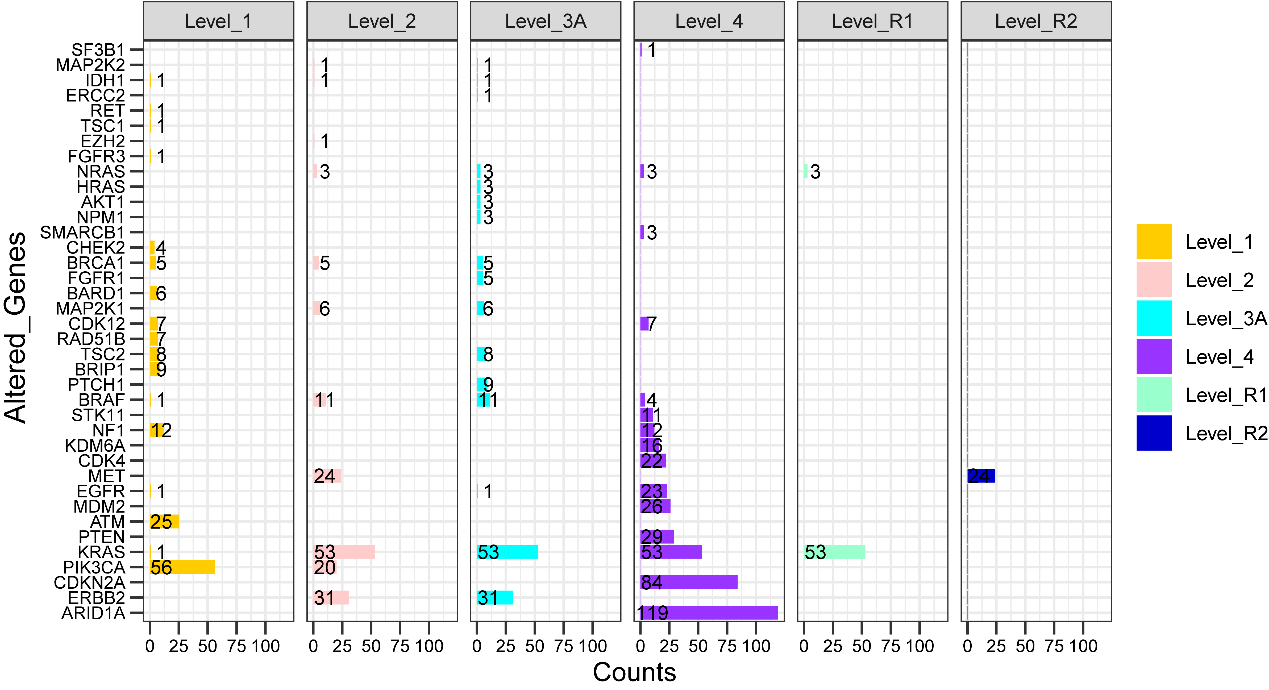


**Supplementary Fig. S3 Distribution of therapeutic levels in altered genes among the subgroups.**
